# Vocabulary Abilities and Parents’ Emotional Regulation Predict Emotional Regulation in School-Age Children but Not Adolescents With and Without Developmental Language Disorder

**DOI:** 10.3389/fpsyg.2021.748283

**Published:** 2021-12-09

**Authors:** Mari Aguilera, Nadia Ahufinger, Núria Esteve-Gibert, Laura Ferinu, Llorenç Andreu, Mònica Sanz-Torrent

**Affiliations:** ^1^Departament de Cognició, Desenvolupament i Psicologia de l’Educació, Secció Cognició, Universitat de Barcelona, Barcelona, Spain; ^2^Institut de Neurociències, Universitat de Barcelona, Barcelona, Spain; ^3^NeuroDevelop eHealth Lab, eHealth Center, Universitat Oberta de Catalunya, Barcelona, Spain; ^4^Estudis de Psicologia i Ciències de l’Educació, Universitat Oberta de Catalunya (UOC), Barcelona, Spain

**Keywords:** emotional regulation, developmental language disorder (DLD), specific language impairment (SLI), expressive vocabulary, receptive vocabulary, parents, children, adolescents

## Abstract

A comprehensive approach, including social and emotional affectations, has been recently proposed as an important framework to understand Developmental Language Disorder (DLD). There is an increasing considerable interest in knowing how language and emotion are related, and as far as we know, the role of the emotional regulation (ER) of parents of children with and without DLD, and their impact on their children’s ER is still unknown. The main aims of this study are to advance our knowledge of ER in school-age children and adolescents with and without DLD, to analyze the predictive value of expressive and receptive vocabulary on ER in school-age children and adolescents, and to explore parental ER and their effect on their children’s and adolescents’ ER. To cover all objectives, we carried out three studies. In the first and second study, expressive and receptive vocabulary were assessed in wave 1, and ER (Emotional Regulation Checklist -ERC- for children and Emotion Regulation Scale -DERS- for adolescents) was assessed in wave 2, 4 years later. Participants in the first study consisted of two groups of school-aged children (13 had DLD and 20 were typically developing children -TD). Participants in the second study consisted of two groups of adolescents (16 had DLD and 16 were TD adolescents). In the third study, the ER of 65 of the parents of the children and adolescents from study 1 were assessed during wave 2 *via* self-reporting the DERS questionnaire. Results showed no significant differences in ER between DLD and TD groups neither in middle childhood nor in adolescence. Concerning vocabulary and ER, expressive language predicted ER in school-age children but not in adolescents. Finally, parental ER explained their school-age children’s ER, but this was not the case in adolescents. In conclusion, the present data indicated that expressive vocabulary has a fundamental role in ER, at least during primary school years, and adds new evidence of the impact of parents’ ER upon their children’s ER, encouraging educators and speech language pathologists to include parents’ assessments in holistic evaluations and interventions for children with language and ER difficulties.

## Introduction

Developmental Language Disorder (DLD), previously known as Specific Language Impairment (SLI), is a neurodevelopmental disorder that affects about 7.5% of the general population (Norbury et al., 2016). Children with DLD have severe and persistent difficulties, to different degrees, in both language production and comprehension in the absence of intellectual disability, hearing loss, or other medical conditions or syndromes known to cause language disorders (Bishop et al., 2016, 2017). Although much of the research into DLD has focused on preschool and school-aged children, studies now show that DLD persists into adolescence and adulthood (Tomblin et al., 1992; Durkin and Conti-Ramsden, 2007; Catts et al., 2008; Conti-Ramsden et al., 2013).

Although there is a consensus that most difficulties in children with DLD are presented in syntax and morphology (Van Der Lely, 1998; Van Der Lely et al., 2004; Moscati et al., 2020), other components such as vocabulary difficulties are also well-defined in this population. In fact, different studies have shown difficulties in the different skills needed to learn new vocabulary such as the ability to store and retrieve new words. For example, children with DLD use high frequency verbs and nouns more often than children with normal language abilities (Eyer and Leonard, 1995; Leonard, 1995) and have more reduced vocabularies than expected for their age (Rice et al., 1990). They score lower in vocabulary tests (Gray et al., 1999) and show less ability for receptive word learning in naturalistic contexts (Rice et al., 1994). Also, it has been shown that they have a reduced sensitivity to the phonological and semantic features of words (Alt and Plante, 2006) and have difficulties mapping labels to new objects (Gray, 2004; Alt and Plante, 2006; Jackson et al., 2019; Ahufinger et al., 2021).

The term SLI has been historically used to refer to children with deficits that are specific to the “language” system (Adani et al., 2014) following the most extensively used diagnostic exclusionary criteria, based on the assumption that language difficulties cannot be explained neither by intellectual disability or other conditions, such as hearing loss, sensory impairment or medical or neurological conditions, such as autism or William’s syndrome, nor by environmental weakness or emotional impact and disturbances (Leonard, 2014; Bishop et al., 2017).

In contrast, the most updated conceptualization of this disorder is based on a broader interpretation of the term to denote the presence of both language-based deficits as well as weaknesses in areas that go beyond language (Tager-Flusberg and Cooper, 1999; Bishop et al., 2016, 2017) due to the increasing number of studies interested in showing the degree to which the deficits in this population are specific to the language system or extend to non-linguistic aspects such us cognition (Kapa and Plante, 2015), memory (Ahufinger et al., 2021), academic achievement (Beitchman et al., 1996; Aguilar-Mediavilla et al., 2019), social interactions (Fujiki et al., 1999; Durkin and Conti-Ramsden, 2007) and emotional abilities (Fujiki et al., 2002, 2004). Consistent with the central tenants of a broad characterization of the disorder and the terminological and conceptualization shift proposed by the CATALISE Consortium, some researchers and clinical professionals are shifting to the term DLD to refer to children who fall into the broader definition of SLI (Lee and Tomblin, 2012; Bishop et al., 2016, 2017). Thus, this broad definition of DLD accepts the diagnostic of DLD in co-occurrence with other disorders such as attentional and hyperactivity disorder (ADHD), developmental coordination disorder (DCD), developmental dyslexia, speech language disorder or emotional disorders. In keeping with this trend, we use the term DLD in this manuscript to refer to this more broadly defined group of children with language-based deficits.

In order to properly communicate with others, it is essential to use abilities such as speech and language, that are related to non-verbal, and verbal means of conveying information and emotions. In turn, emotional competence, which refers to the ability to understand, express, and regulate emotions (Denham, 1998), is important for school adjustment, social functioning, and success. In this regard, language skills have been proposed to be basic to social and emotional development in children and adolescents (Kopp, 1989, 1992; Eisenberg et al., 2005). In the same line, Saarni (1999) proposed that language ability promotes representation of emotional experiences, facilitating their elaboration and comprehension and *regulation* of emotions.

It is generally accepted that language abilities have a positive influence on emotional competence (Beck et al., 2012). Notably, vocabulary abilities play a crucial role in children’s emotional abilities because it gives the possibility to label emotions, making them explicit and communicable (Barrett et al., 2007; Cole and Cohen, 2009) and facilitates the capacity to represent feelings states and to enhance perspective-taking (e.g., Pons et al., 2003; Downs et al., 2007; Cole et al., 2010; Köckeritz et al., 2010). In this regard, different studies have shown a strong relation between children’s vocabulary abilities and different aspects of emotional competence. For example, Cutting and Dunn (1999) found a relationship between emotion understanding (facial emotion recognition and emotion situation knowledge) and receptive vocabulary in 4-year-old toddlers. In addition, Bosacki and Moore (2004) showed a positive and strong relationship between knowledge of basic (happy, sad) and complex emotions (proud, embarrassed) and receptive vocabulary in 3-year-old children. Downs et al. (2007) and Köckeritz et al. (2010) also demonstrated positive associations between preschoolers’ receptive vocabulary and more sophisticated aspects of emotion understanding, such as the understanding of complex emotions.

Recently, emotional regulation (ER) has emerged as a novel construct in the field of emotion (Eisenberg et al., 2004; Gross, 2015b). ER is an essential core of emotional competence, and refers to a human ability that allows modification of the quality, intensity, duration and expression of emotions according to the goals that one is intending to achieve (Gross, 2015a). Thus, ER involves consciously, or not consciously, managing emotions and their expression (Eisenberg et al., 2005). For example, when people pay attention to social media in order to decrease their anger after an argument or when someone needs to decrease the expression of joy when faced with a sad friend. In early stages of development, the extrinsic emotion process derived from the caregiver’s intervention becomes crucial for the development of ER. For example, extrinsic ER is carried out in a situation when parents show and play with their keys when their baby is irritated, in an attempt to focus the baby’s attention and to calm them down. Another example is when parents talk to their children about feelings after an argument. Even though, after the first stages of infancy, intrinsic ER, that is, the process of regulating an emotion in one’s self, increasingly supplements extrinsic mechanisms (Thompson, 1991).

Interestingly, recent research suggested that women used more types of ER strategies, and are more flexible in using them, in different contexts (Goubet and Chrysikou, 2019). These results are consistent with previous research indicating women have greater ER skills from childhood than men (see Nolen-Hoeksema, 2012 for review).

Some studies have shown a specific relationship between general language abilities and ER. In particular, some evidence shows that good oral language skills are associated with higher levels of ER (Cole et al., 2010). Specific to vocabulary, longitudinal studies such as Vallotton and Ayoub (2011) showed that babies’ expressive vocabulary skills (measured and coded with CHILDES as the number of unique vocabulary words spoken during the mother–child observation) positively impacted on their later regulation skills at 36 months of age. In addition, Ornaghi et al. (2019) assessed a large group of Italian toddlers (mean age = 28 months) to study the contribution of emotion knowledge, language ability, and maternal emotion socialization style to ER. They asked the mothers to fill-out both the vocabulary section (i.e., child’s word production) of the McArthur−Bates−Communicative Development Inventories, and the Emotional Regulation Checklist (ERC), a widely used questionnaire developed by Shields and Cicchetti (1997) that assesses the dimensions of positive ER and negativity (affective lability, intensity, valence, flexibility, and situational appropriateness). They found that toddlers’ expressive vocabulary score was significantly positively associated with their ER.

It is particularly interesting to study the relationship between ER in children with atypical language development, such as the population with DLD, because a clinical evidence of the connection between DLD and emotional difficulties has been demonstrated. A growing body of research have shown higher levels of emotional difficulties in population with DLD compared to their typically developing peers in childhood (for review see Yew and O’Kearney, 2013) and adolescence (Durkin and Conti-Ramsden, 2010; St Clair et al., 2011; Conti-Ramsden et al., 2013) or in adulthood (Botting et al., 2016). The symptomatology most described is related to an increase of feelings of anxiety and depression compared to their typically developing (TD) peers (Beitchman et al., 2001; Conti-Ramsden and Botting, 2008; St Clair et al., 2011; van den Bedem et al., 2018; Forrest et al., 2021). Moreover, different authors have studied the trajectory of emotional difficulties throughout different developmental stages, showing heterogeneous emotional DLD trajectories during a lifetime (Beitchman et al., 2001; Redmond and Rice, 2002; Conti-Ramsden et al., 2019; St Clair et al., 2019).

Limited studies have specifically studied ER in DLD. Fujiki et al. (2002, 2004) conducted a series of studies assessing ER in a group of 5- to 13-year-old children with DLD and a group of typically developing (TD) children matched by age and sex in measures of ER derived from a composite score of the ERC questionnaire. Both studies showed a greater difficulty to regulate their emotions in the DLD group compared to their TD peers. More specifically, children with DLD presented significantly lower scores compared to their TD peers in the ER subscale that assess emotional awareness and appropriate display of emotions and the identification of others’ emotional states, compared to other subscales such as the lability/negativity subscale that assesses difficulties in regulating emotions, variability of mood and inflexibility of ER. Regarding age differences (6–9 years to 10–13 years), non-significant main effects were reported concerning ER, but girls presented better ER than boys, independently of whether they had DLD or not. Moreover, these studies indicated that both ER and language level (overall lexical/semantic, syntactic, supra-linguistic and pragmatic score) had a strong effect on social behavior skills (Fujiki et al., 2004). Recent findings by Forrest et al. (2020) also suggest poor ER in children with DLD. In addition they found that ER in both children with and without DLD became a strong predictor of emotional difficulties during childhood (Forrest et al., 2020). Additionally, van den Bedem et al. (2018) found that avoidant ER strategies (ignoring or distracting for the situation to diminish the negative impact of the situation) in children with and without DLD are strongly related with later development of depressive symptoms. Further studies on DLD and ER are needed to corroborate those previous results.

Parenting, in addition to language abilities, is also a key factor in the development of ER (Morris et al., 2007; Baker, 2018). Parenting can influence ER through three factors proposed by Morris et al. (2007): observational learning (observing parents while interacting with their own emotions), parenting practices (facilitating or hindering emotional identification and understanding of child emotions) and emotional climate (involvement quality of familiar relationship). Some longitudinal studies have pointed to the longstanding effect of parenting on child ER, parenting assessed in terms of attachment or quality of the parent–child relationship (Hilt et al., 2012; Girme et al., 2020). Furthermore, a study by Tammilehto et al. (2021), concerning parenting (parental autonomy and intimacy) and ER, found that the effect of parenting quality on childhood has no lasting effect on adolescent ER patterns. Taken together, these studies usually included measures of parent–child attachment or quality of parenting but did not include measures of ER in parents. Moreover, most of these research only focused on the mother’s effect on ER, and did not include fathers or did not report on them, although both are expected to influence their children’s ER (Van Lissa et al., 2019). Despite this interest, to the best of our knowledge, no studies have explored the putative role of parental ER on their children’s or adolescents’ ER with and without DLD.

Another interesting point of view is the impact of having a daughter or a son with DLD in the parents’ ER. Recently, a study carried out by Ash et al. (2020), interviewing mothers of children with language disorders, showed that mothers reported additional distress due to language problems, but they also had intense emotional experiences due to feeling responsible for, and guilty about, the language problems of their child, as a result they questioned themselves regarding their parenting skills, and also pointed out the distress surrounding the diagnostic processes and the academic future and social performance of their children. Moreover, higher levels of maternal stress had also been described (Lisa et al., 2019). Hence, motherhood of children with DLD is characterized by increasing depressive and anxiety symptomatology (Von Suchodoletz and Macharey, 2006). As far as we are concern there are no studies that compare the ER of parents whose children present DLD to the ER of parents with children with normal language abilities. The aim of this project is four-fold: first, to examine whether children with DLD have more ER difficulties compared to their TD peers and to explore this pattern in adolescents with DLD; second, to investigate if receptive and expressive vocabulary at younger ages can predict the ER of school-aged children with and without DLD 4 years later; third, to investigate if this temporal relation between vocabulary skills and ER in children with and without DLD exists in adolescents; fourth, to explore whether the ER of the parents whose children present DLD differs from the ER of parents with TD children, and whether parental ER is related to the ER of their children and adolescents (with and without DLD). We also considered the differences between sexes in ER as a secondary goal since past studies have found differences between males and females in ER abilities. In addition, including sex difference analyses is important in all human studies because it is a factor that can influence all stages of research or development processes, from strategic considerations for establishing priorities and building theory to the more routine tasks of formulating questions, designing methodologies, and interpreting data (Schiebinger et al., 2011). Finally, it is important to note that almost all the studies that assess emotional difficulties and ER specifically in DLD have been run with English-speaking population and this is the first study that addresses ER in bilingual Catalan-Spanish children with and without DLD.

## Study 1

This study was approved by the Universitat Oberta de Catalunya (UOC) Ethics Committee. The study was performed in accordance with the ethical standards laid out in the 1964 Declaration of Helsinki and subsequent updates (WMA, 2013).

Study 1 was carried out to examine differences in ER of a group of school-age children with DLD and a group of TD children. Also, we investigated whether children’s expressive and receptive vocabulary in wave 1, when they were around 7-years-old, predicted ER in wave 2, when they were around 11-years-old.

### Methods

In study 1 a two-wave 4-year-lag longitudinal study was carried out with a total of 33 Catalan-Spanish speaking children (11 girls and 22 boys) participating: 13 children with Developmental Language Disorder (Age_*mWAVE*1_ = 7.12; Age_*mWAVE*2_ = 10.98; 5 girls and 8 boys) and 20 typically developing (TD) children (Age_*mWAVE*1_ = 7.52; Age_*mWAVE*2_ = 10.67; 6 girls and 14 boys).

#### Wave 1

##### Participants

The participants were part of a larger study conducted in 2017 in Barcelona, Spain [a detailed description can be found elsewhere (Ahufinger et al., 2021)]. Wave 1 was carried out in 2017, where Children with DLD were recruited from institutions, organizations, and schools around Catalonia and were identified with the help of the Catalan Center of Resources for Hearing-Impaired People (CREDA), members of the Catalan service for school counseling and guidance (EAP) and Catalan Association of Specific Language Impairment (ATELCA), that work in conjunction with public and private schools throughout Catalonia to identify children with DLD or children with language difficulties. The TD children were recruited from public schools within the Barcelona greater metropolitan area. In wave 1, information about the study was provided for the parents and, once they agreed to participate, they signed the informed consent. It consisted of two sessions of approximately 90 min each, with the same evaluators.

All participants met the following inclusion criteria: (a) non-verbal intellectual quotient (NVIQ) ≥ 70; (b) normal hearing at 500, 1,000, 2,000, and 4,000 Hz at 20 dB based on the American National Standard Institute (1997); (c) normal or corrected-to-normal vision, (d) normal oral and speech motor abilities by a certified Speech Language Pathologist; and (e) were native bilingual Catalan-Spanish speakers. Children were excluded if parents reported: (a) other biomedical conditions commonly linked to genetic or neurological causes such as Autism, intellectual disability or Down and Williams syndromes (Bishop et al., 2017) (b) frank neurological signs or, (c) seizure disorders or use of medication to control seizures.

##### Instruments and procedure

The children in the DLD group had a formal diagnosis of language impairment or were in the process of being diagnosed and were receiving speech language services at the time of the study. The TD children were at grade level in school, had no history of, nor diagnosis of, language learning disability, and had never received speech and language services. To confirm participant’s language status, standardized testing was completed by two trained researchers at the time of the study and included the Non-verbal IQ (NVIQ) Kaufman Brief Intelligence Test (K-BIT, Kaufman and Kaufman, 1990), Clinical Evaluation of Language Fundamentals – Fourth Edition, Spanish (CELF-4-Spanish; Semel et al., 2006) (1) Core Language score, (2) Expressive Language score, and/or (3) Receptive Language score. For the children with DLD, either Core, Receptive or Expressive CELF composite scores were 1 *SD* or more below age-level expectations. For the children in the TD group, CELF composite scores were all at or above age-level expectations.

The two groups (DLD and TD) did not differ in age or sex distribution (see Table 1). Sociodemographic characteristics (parental education and economic status) for both groups are also reported in Table 1.

**TABLE 1:** Socio-demographic characteristics of the children with Developmental Language Disorder (DLD) and the typically developing (TD) children (Study 1).

	DLD (*n* = 13)	TD (*n* = 20)	*Statistic*	*p*-value
Age in months (wave 1)	88.85 (±11.45)	96.70 (±13.02)	1.77 (t)	0.086
Age in months (wave 2)	137.46 (±11.63)	134.00 (±12.33)	−0.81 (t)	0.427
Sex distribution			0.25 (χ^2^)	0.614
Boys	8 (61.5%)	14 (70%)		
Girls	5 (38.5%)	6 (30%)		
Mother’s education level			0.39 (χ^2^)	0.820
Elementary school or high school certificate	2 (15%)	2 (10%)		
Bachelor’s degree or similar	7 (54%)	10 (50%)		
Higher undergraduate/master’s degree	8 (31%)	4 (40%)		
Father’s education level			0.81 (χ^2^)	0.667
Elementary school or high school certificate	2 (20%)	4 (22.2%)		
Bachelor’s degree or similar	5 (50%)	6 (33.3%)		
Higher undergraduate/master’s degree	3 (30%)	8 (44.4%)		
Economic status			4.3 (χ^2^)	0.114
Less than 15,000€/year	4 (31%)	3 (15%)		
From 16,000 to 35,000€/year	6 (46%)	5 (25%)		
More than 35,000€/year	3 (23%)	12 (60%)		

In addition, to investigate whether children’s expressive and receptive vocabulary in wave 1 predicted ER in wave 2, all children also completed the Peabody Picture Vocabulary Test, Third Edition, Spanish version (PPVT-III; Dunn et al., 2006), and the expressive vocabulary subtest of the Spanish version of the Kaufman Brief Intelligence test (K-BIT-Voc; Kaufman and Kaufman, 2004). For the PPVT-III, the examiner presents a series of pages that contain four pictures and says a word. The child must identify (say the number that corresponds to the picture or point to the picture) the picture that best corresponds to the word. For the subtest K-Bit Vocabulary children up to 8 years of age only must perform the “vocabulary task” that consists of orally naming the drawing-objects that the experiment presents one by one in a series of pages. Children older than 8 must perform the “vocabulary task” and also the “Definition task” that involves guessing words using two clues: a definition of the word and some letters contained in the word to guess. For example, the examiner says, “Place with plants and flowers” and shows the child a page with the following cue: G_ _ D _ _. It is expected for the child to say: “Garden.”

Language and cognitive assessment at wave 1 are shown in Table 2. Non-verbal IQ was within normal limits in both groups. There were significant statistical differences between DLD and TD groups in the three language scales in CELF-4 and in the expressive and receptive vocabulary scores.

**TABLE 2:** Standardized scores for language, cognitive assessment measures for children with Developmental Language Disorder (DLD) and typically developing (TD) children measured at wave 1 (Study 1).

	DLD (*n* = 13)		TD (*n* = 20)		Comparison	
Variable	Mean	SD	Mean	SD	t	*p*
K-BIT mat (IQ)^a^	102.46	8.72	104.20	10.02	0.51	0.613
CELF- CLS^b^	74.08	12.37	109.60	7.68	10.21	<0.001
CELF- ELS^c^	74.31	10.34	108.80	9.39	9.91	<0.001
CELF –RLS^d^	79.46	14.08	105.10	7.43	6.04	<0.001
K-BIT Voc^e^	84.69	13.06	105.70	10.66	5.06	<0.001
PPVT_III^f^	85.62	16.50	103.55	12.36	3.57	0.001

*^a^K-BIT IQ. Kaufman Brief Intelligence: Non-verbal intelligence score. Scaled scores (M = 100, SD = 15).*

*^b^CELF-4 CLS = Spanish Clinical Evaluation of Language Fundamentals, 4th Edition: Core Language score. Scaled scores (M = 100, SD = 15).*

*^c^CELF-4 ELS = Spanish Clinical Evaluation of Language Fundamentals, 4th Edition: Expressive Language score. Scaled scores (M = 100, SD = 15).*

*^d^CELF-4 RLS = Spanish Clinical Evaluation of Language Fundamentals, 4th Edition: Receptive Language score. Scaled scores (M = 100, SD = 15).*

*^e^K-BIT vocabulary. Kaufman Brief Intelligence: Expressive vocabulary score. Scaled scores (M = 100, SD = 15).*

*^f^PPVT-III = Peabody Picture Vocabulary Test, Third Edition. Scaled scores (M = 100, SD = 15).*

#### Wave 2

##### Participants

Families of the 33 children with and without DLD that participated in wave 1 were asked to participate 4 years later, in 2021, to assess ER. The study was carried out by the same research group in 2017 and 2021. Due to the COVID-19 global pandemic, all families were asked to answer an online questionnaire (available from February to April 2021) that included ER assessment, and they signed a new consent form.

##### Instruments and procedure

To assess ER in school-aged children, a Spanish translation of the Emotional Regulation Checklist (ERC, Shields and Cicchetti, 1997; Sarmento-Henrique et al., 2017) was used. It is a list of 24 items that measure affective lability, intensity, valence, flexibility, and situational appropriateness that must be completed by the parents. Each item is rated on a 4-point Likert scale (1 never and 4 almost always). An ERC composite score is generated where higher values on the index show more difficulties in ER. In addition, the questionnaire extracts two subscales: The *Emotion Regulation* subscale (ER subscale) that includes items describing situationally appropriate affective displays, empathy, and emotional self-awareness, and the *Lability/Negativity* subscale (Lab subscale) that is comprised of items representing a lack of flexibility, mood lability, and dysregulated negative affect. Strong evidence for validity and reliability has been reported for composite the ERC and both subscales (Shields and Cicchetti, 1997). In our study, Cronbach’s alfa was 0.75 in the ER subscale, and 0.76 for Lab subscale. For composite ERC subscale a 0.78 the Cronbach’s alfa was found for 23 items (item 12 did not load in any subscale).

### Results

#### Differences in Emotional Regulation Between School-Aged Children With and Without Developmental Language Disorder

Due to the small sample size (<30 in each group) and considering violations of the equal variance assumption (Levene test *p* = 0.011) and deviation from normality (Shapiro-Wilk *p* = 0.009) in the ER subscale in the TD group, non-parametric Mann–Whitney *U* was performed to test differences between groups regarding ER assessed by the ERC. Non-significant differences were found between children with DLD and TD in none of the two subscales (*ER* subscale and *Lab* subscale), or the ERC composite score. Numerical differences (not significant) between groups (see Table 3) were in the direction of our hypothesis (DLD group less ER than TD group) and medium effect sizes were found in ER score (Lab subscales = 0.312 and −0.300, respectively) and in the ERC composite score (−0.335). No sex differences were found for ER, neither when language groups were taken into account (DLD or TD) (boys *n* = 22; girls *n* = 11, ERC composite: Mdn boys = 40.00, Mdn girls = 38.00, *p* = 0.646; *ER* subscale: Mdn boys = 28.00, Mdn girls = 26.00, *p* = 0.225; *Lab* subscale: Mdn boys = 26.50, Mdn girls = 27.00, *p* = 0.924) nor when language groups were considered (see Supplementary Table 1).

**TABLE 3:** Emotion regulation for children with Developmental Language Disorder (DLD) and typically developing (TD) children measured at wave 2 (Study 1).

	DLD (*n* = 13)			TD (*n* = 20)				Comparison	
Variable	Mean	*SD*	Median MAD	Mean	*SD*	Mean MAD	*Z* ^a^	Effect size
ER subscale	24.92	4.11	26.00	27.40	2.70	28.00	−1.50	0.31
			4.00			1.50		
Lab subscale	29.46	6.09	28.00	26.50	4.68	25.50	−1.44	−0.30
			4.00			3.00		
Composite ERC	44.54	9.35	43.00	39.10	6.23	38.00	−1.61	−0.34
			6.00			5.00		

*ER subscale, Emotion Regulation Subscale from ERC; Lab Subscale, Lability/Negativity Subscale from ERC; ERC, emotion regulation checklist.*

*^a^Non-parametric Mann–Whitney U test was performed; For the Mann–Whitney test, effect size is given by the rank biserial correlation.*

#### Relationship Between School-Aged Children’s Vocabulary Knowledge and Emotional Regulation

We conducted multiple regression analyses for all school-aged children (DLD and TD) to investigate whether expressive and receptive vocabulary assessed during wave 1 were significant predictors for ER in wave 2.

We considered three predictive models using ER as a predicted variable. The ER subscale was significantly correlated with expressive vocabulary (*r* = 0.35, *p* < 0.05) but not receptive vocabulary (*r* = 0.12, *p* < ns), nor age (*r* = −0.19, *p* < ns) or NVIQ (*r* = 0.08, *p* < ns). The Lab subscale was not correlated with expressive vocabulary (*r* = −0.26, *p* < ns), nor receptive vocabulary (*r* = −0.04, *p* < ns), nor age (*r* = 0.16, *p* < ns) or NVIQ (*r* = 0.01, *p* < ns). Finally, the ERC composite was significantly correlated with expressive vocabulary (*r* = −0.35, *p* < 0.05) but not receptive vocabulary (*r* = −0.11, *p* < ns) age (*r* = 0.22, *p* < ns) nor NVIQ (*r* = −0.03, *p* < ns). For the three regression models, we used forward selection in the independent variables. Age (in wave 2) and NVIQ (wave 1) were introduced as putative predictors in the model in addition to expressive and receptive vocabulary (wave 1) because we considered them as control variables. In the first model, the dependent variable was the ER subscale (in wave 2); in the second model, the dependent variable was the Lab subscale; in the third model, the dependent variable was the ERC composite score. In the first model, expressive vocabulary was the only variable included in the regression model and explained 16.2% of the variability in ER subscale as indicated by the significant *B* coefficient, *St.*β = *0.402*; *t* = 2.44; *F* = 5.975, *p* = *0.020* (Table 4). The second model did not retain any predictors for the Lab subscale. Finally, in the third model expressive vocabulary was the only variable included and predicted 12.4% of variability in the ERC composite score (*B* coefficient, *St.*β = −*0.352*; *t* = −2.09; *F* = 4.387, *p* = 0.044) (see Table 4). In both significant models, all the assumptions for regression analyses were satisfied, including independence of errors, homoscedasticity, and normality of the distribution of residuals.

**TABLE 4:** Regression model: influences of expressed and receptive vocabulary at wave 1 on emotional regulation in children at wave 2 (Study 1).

	K-BIT Voc^a^		
Variable	St. β	*t*	CI
ER subscale^a^	0.402 *R* = 0.162	2.444*	0.015, 0.16
Composite ERC^a^	−0.352	−2.095*	−0.356, −0.05
	*R* = 0.124		

^a^ *Forward selection excluded non-verbal IQ Kaufman Brief Intelligence (K-BIT mat), age and Peabody Picture Vocabulary Test (PPVT-III) from the model; ERC, emotion regulation checklist.*

Lab subscale as dependent variable forward selection excluded all variables from the model (K-BIT mat, age, K-BIT voc and PPVT-III). *p ≤ 0.05.

The direction of the relationship was positive in the first model and negative in the third model. That means participants with higher scores in expressive vocabulary obtained higher scores in the ER subscale (higher scores indicate better ER) and obtained lower values in the ERC composite score (higher values in the index show more difficulties in ER). Thus, higher expressive vocabulary in wave 1 predicted better ER functioning in wave 2 in school-aged children under 12-years-old.

## Study 2

This study was approved by the Universitat Oberta de Catalunya (UOC) Ethics Committee. The study was performed in accordance with the ethical standards laid out in the 1964 Declaration of Helsinki and subsequent updates (WMA, 2013).

Study 2 was carried out to examine differences in ER of a group of adolescents with DLD and a group of TD adolescents. Also, we investigated whether their expressive and receptive vocabulary in wave 1, when they were around 11-years-old, predicted ER in wave 2, when they were around 15-years-old.

### Methods

As in study 1, the participants were part of a larger study conducted in 2017 in Barcelona, Spain (for details see Ahufinger et al., 2021), through which a two-wave 4-year-lag longitudinal study was carried out. In study 2 a total of 32 Catalan-Spanish speaking adolescents (11 girls and 21 boys), 16 adolescents with DLD (Age_*mWAVE*1_ = 11.05; Age_*mWAVE*2_ = 15.12; 7 girls and 9 boys) and 16 TD adolescents (Age_*mWAVE*1_ = 10.99; Age_*mWAVE*2_ = 14.17; 4 girls and 12 boys) participated.

#### Wave 1

##### Participants

Recruitment procedure, criteria, and instruments to assess adolescents were carried out in the same way described in Study 1. The two groups (DLD and TD) did not differ in mean age in months at wave 1, but DLD at wave 2 presented higher mean age in months compared with TD adolescents. There were no differences in the sex distribution (see Table 5). Sociodemographic characteristics (parental education and economic status) for both groups are also reported in Table 5.

**TABLE 5:** Socio-demographic characteristics of the adolescent with DLD and the typically developing (TD) adolescent (Study 2).

	DLD *n* = 16	TD *n* = 16	*Statistic*	*p*-value
Age in months (wave 1)	137.56 (±20.19)	136.50 (±13.84)	−0.17 (t)	0.863
Age in months (wave 2)	186.75 (±20.00)	173.81 (±13.19)	−2.16 (t)	0.039
Sex distribution			1.24 (χ^2^)	0.264
Boys	9 (56%)	12 (75%)		
Girls	7 (44%)	4 (25%)		
Mother’s education level			1.23 (χ^2^)	0.541
Elementary school or high school certificate	3 (20%)	2 (12.5%)		
Bachelor’s degree or similar	9 (60%)	8 (50%)		
Higher undergraduate/master’s degree	3 (20%)	6 (37.5%)		
Father’s education level			1.92 (χ^2^)	0.383
Elementary school or high school certificate	4 (31%)	2 (14%)		
Bachelor’s degree or similar	7 (54%)	7 (50%)		
Higher undergraduate/master’s degree	2 (15%)	5 (36%)		
Economic status			2.91 (χ^2^)	0.233
Less than 15,000€/year	4 (27%)	1 (6%)		
From 16,000 to 35,000€/year	6 (40%)	6 (38%)		
More than 35,000€/year	5 (33%)	9 (56%)		

As in Study 1, in addition to the language and cognitive assessment applied to ensure the diagnostic criteria of language group/profile, all participants also completed the Peabody Picture Vocabulary Test, Third Edition, Spanish version (PPVT-III; Dunn et al., 2006) and the expressive vocabulary subtest of the Spanish version of the Kaufman Brief Intelligence test (K-BIT-Voc; Kaufman and Kaufman, 2004) to investigate whether participants’ expressive and receptive vocabulary at wave 1 predicted ER at wave 2.

Language and cognitive assessment at wave 1 are shown in Table 6. The overall mean score for both groups were within the normal range. There were statistical differences on the three language scales in CELF-4 and, expressive and receptive vocabulary scores were statistically significantly different between DLD and TD groups.

**TABLE 6:** Standardized scores for language, cognitive assessment for adolescent with Developmental Language Disorder (DLD) and typically developing (TD) adolescent at wave 1 (Study 2).

	DLD (*n* = 16)		TD (*n* = 16)		Comparison	
Variable	Mean	*SD*	Mean	*SD*	*t*	*p*
K-BIT mat (IQ)^a^	91.88	13.41	104.13	11.17	2.81	0.009
CELF- CLS^b^	80.31	11.25	109.87	4.03	9.89	<0.001
CELF- ELS^c^	80.19	8.32	109.69	5.71	11.69	<0.001
CELF –RLS^d^	82.63	10.59	109.13	7.92	8.02	<0.001
K-BIT Voc^e^	75.94	10.18	99.00	9.98	6.47	<0.001
PPVT_III^f^	83.50	18.76	102.63	14.99	3.19	0.003

*^a^K-BIT IQ. Kaufman Brief Intelligence: non-verbal intelligence score, scaled scores (M = 100, SD = 15).*

*^b^CELF-4 CLS = Spanish Clinical Evaluation of Language Fundamentals, 4th Edition: Core Language score. Scaled scores (M = 100, SD = 15).*

*^c^CELF-4 ELS = Spanish Clinical Evaluation of Language Fundamentals, 4th Edition: Expressive Language score. Scaled scores (M = 100, SD = 15).*

*^d^CELF-4 RLS = Spanish Clinical Evaluation of Language Fundamentals, 4th Edition: Receptive Language score. Scaled scores (M = 100, SD = 15).*

*^e^K-BIT vocabulary. Kaufman Brief Intelligence: Expressive vocabulary score. Scaled scores (M = 100, SD = 15).*

*^f^PPVT-III = Peabody Picture Vocabulary Test, Third Edition. Scaled scores (M = 100, SD = 15).*

#### Wave 2

##### Participants

As in Study 1, families of the 32 adolescents with and without DLD that participated in wave 1 were asked to participate 4 years later, in 2021 to assess the adolescents’ ER. The study was carried out by the same research group in 2017 and 2021. Like in study 1 all families signed a new consent form, but now they authorized their sons and daughters to answer the online ER questionnaire themselves (available from February to April 2021). Families were asked to ensure that adolescents were in a quiet room when answering the questionnaire, and they were asked to let their children answer with adult help if necessary to ensure reading comprehension.

##### Instruments and procedure

To asses adolescents’ ER, the self-reported Difficulties in Emotion Regulation Scale (DERS; Gratz and Roemer, 2004) was used. The Spanish version was available for adolescents (Gómez-Simón et al., 2014). In this scale participants are asked to indicate how often the items apply to themselves, ranging from 0 (almost never) to 5 (almost always). A composite DERS score was calculated thought the sum of all items, higher scores indicating higher difficulties in emotion regulation. Six subscales can be derived from this scale: awareness and understanding of emotions (Awareness subscale), clarity of your own emotions (Clarity subscale), level of acceptance of emotions (Non-acceptance subscale), level of control of own behavior when experiencing negative emotions (Impulse subscale), the ability to maintain goal-directed behaviors when experience negative emotions (Goals subscale) and the level of access of effective emotion regulation strategies (Strategies subscale). Good psychometric properties were reported in the adolescent version (Gómez-Simón et al., 2014). Cronbach’s alfa was 0.86 for the composite DERS score, and Cronbach’s alpha internal reliability values in subscales ranged from α = 0.54 (Strategies subscale) to α = 0.88 (Goals subscale).

### Results

#### Differences in Emotional Regulation Between Adolescents With and Without Developmental Language Disorder

A non-parametric test was used due to the small sample size (<30 in each group) and considering violations of the equal variance assumption (through Levene test) in some data in both DLD or TD groups, or one of them, and deviation from normality (Shapiro–Wilk) were found in some variables in both groups, or one of them. A Non-parametric Mann–Whitney *U* revealed non-statistically significant differences between DLD and TD groups in emotion regulation in none of the measures (neither in the DERS composite score nor in any of the six subscales, see Table 7). Numerical differences (not significant) between groups (see Table 3) were in the direction of our hypothesis (DLD group less ER than TD group), and effect sizes revealed that these differences were small [except for the Awareness subscale, for which the effect size was medium (−0.355)]. No sex differences were found in adolescent emotional regulation, neither when both language groups were considered together (DLD or TD) (boys *n* = 21; girls *n* = 11; DERS composite: Mdn boys = 47.00, Mdn girls = 52.00, *p* = 0.462; Awareness: Mdn boys = 10.00, Mdn girls = 11.00, *p* = 0.489; Impulse: Mdn boys = 6.00, Mdn girls = 7.00, *p* = 0.984; Non-acceptance: Mdn boys = 8.00, Mdn girls = 11.00, *p* = 0.557; Goals: Mdn boys = 8.00, Mdn girls = 8.00, *p* = 0.841; Clarity: Mdn boys = 7.00, Mdn girls = 7.00, *p* = 0.482; and Strategies: Mdn boys = 5.00, Mdn girls = 5.00, *p* = 0.231), nor when DLD and TD group were analyzed separately (see Supplementary Table 2).

**TABLE 7:** Emotion regulation for adolescent with Developmental Language Disorder (DLD) and typically developing (TD) adolescent measured at wave 2 (Study 2).

	DLD (*n* = 16)			TD (*n* = 16)			Comparison	
	
Variable	Mean	*SD*	Median MAD	Mean	*SD*	Median MAD	*Z* ^a^	Effect size
Awareness	11.63	3.85	12.50	10.00	2.73	10.00	−1.37	−0.28
			2.50			2.00		
Impulse	9.06	4.73	8.00	6.75	2.57	5.50	−1.52	−0.31
			3.00			0.50		
Non-acceptance	10.75	4.31	8.00	10.13	2.78	9.50	−0.096	0.02
			1.00			1.50		
Goals	10.00	4.80	8.50	7.88	2.83	7.50	−1.19	−0.25
			3.50			1.50		
Clarity	8.13	3.34	7.00	7.00	2.22	7.00	−0.73	−0.15
			2.00			1.00		
Strategies	5.50	1.75	5.00	5.56	1.83	5.00	−0.25	0.05
			1.50			1.00		
Composite DERS	55.06	15.27	51.00	47.31	8.31	48.00	−1.15	−0.24
			13.50			6.50		

*DERS, Difficulties in Emotion Regulation Scale.*

*^a^Non-parametric Mann–Whitney U test was performed; For the Mann–Whitney test, effect size is given by the rank biserial correlation.*

#### Relationship Between Adolescents’ Vocabulary Knowledge and Emotional Regulation

We conducted multiple regression analyses for adolescents to investigate whether expressive and receptive vocabulary assessed in wave 1 were significant predictors for adolescent ER in wave 2. The Impulse subscale was significantly correlated with receptive vocabulary (*r* = −0.39, *p* < 0.05) but not expressive vocabulary (*r* = −0.25, *p* < ns), not age (*r* = 0.15, *p* < ns) or NVIQ (*r* = −0.24, *p* < ns). The rest of subscales and DERS composite scores were not significantly correlated with any of the putative predictors.

We considered 7 models using each of the 7 ER scores of the DERS composite score and subscales as predicted variables (DERS composite score, Awareness, Clarity, Non-acceptance, Impulse, Goals and Strategies subscales). A forward selection in the independent variables was used. Age (wave 2) and NVIQ (wave 1) were introduced as putative predictors in the model in addition to expressive and receptive vocabulary (wave 1) because we considered them control variables. Results showed that regression models using forward selection did not include any putative predictors in any of the models proposed.

## Study 3

This study was approved by the Universitat Oberta de Catalunya (UOC) Ethics Committee. The study was performed in accordance with the ethical standards laid out in the 1964 Declaration of Helsinki and subsequent updates (WMA, 2013).

The aim of study 3 was to explore whether the ER of the parents whose children present DLD differs from the ER of parents with TD children, and whether parental ER is related to the ER of their children and adolescents (with and without DLD).

### Methods

#### Participants

The participants were the 65 parents of the 33 school-aged children and the 32 adolescents with and without DLD that participated in study 1 and study 2. In total there were 29 parents of children with DLD (DLD parents) and 36 parents of TD children (TD parents’ group). Socioeconomical status were described for parents of children in Table 1 and for parents of adolescents in Table 5.

#### Instruments and Procedure

All families were asked to answer a parental online questionnaire (to be filled-out by the mother, the father, or the legal guardian) about emotional self-regulation. As the families decided which parent would answer the questionnaire, the final sample included 54 mothers (24 had a child with DLD and 30 had a TD child) and 11 fathers (5 had a child with DLD and 6 had a TD child). In addition to the questionnaire, parents signed a consent form to participate in the study.

To assess parental ER, they filled-out the adult version of the Spanish self-reported Difficulties in Emotion Regulation Scale (DERS; Gratz and Roemer, 2004; Hervás and Jódar, 2008). DERS characteristics and subscales are detailed in Study 2. Good psychometric properties were reported in the adult version (Hervás and Jódar, 2008). Cronbach’s alfa was 0.93 for the composite DERS score, and Cronbach’s alpha internal reliability values ranged from α = 0.69 (Impulse subscale) to α = 0.90 (Non-acceptance subscale) in subscales.

#### Differences Between the Emotional Regulation of Parents of Children and Adolescents With and Without Developmental Language Disorder

A parametric *T*-test revealed non-statistical differences between the DLD parents’ group and the TD parents’ group in emotion regulation in none of the measures, and Hedges’ g indicated low effect sizes in all variables of parental ER (DERS composite score or the six subscales, see Table 8). No sex differences were found between mothers and fathers on the composite, nor on or any of the DERS subscales (women *n* = 54; men *n* = 11; DERS composite: Mdn women = 43.00, Mdn men = 41.00, *p* = 0.902; Awareness: Mdn women = 8.00, Mdn men = 8.00, *p* = 0.771; Impulse: Mdn women = 6.00, Mdn men = 6.00, *p* = 0.588; Non-acceptance: Mdn women = 10.50, Mdn men = 14.00, *p* = 0.132; Goals: Mdn women = 7.00, Mdn men = 7.00, *p* = 0.880; Clarity: Mdn women = 5.00, Mdn men = 5.00, *p* = 0.957; and Strategies: Mdn women = 5.00, Mdn men = 4.00, *p* = 0.567).

**TABLE 8:** Emotion regulation scores for parents of children and adolescents with Developmental Language Disorder (DLD) and parents of a typically developing (TD) children and adolescents (Study 3).

	DLD parents		TD parents		Comparison	
	(*n* = 29)		(*n* = 36)		Comparison	
Variable	Mean	*SD*	Mean	*SD*	*T*	*p* Hedges’ *g*
Awareness	8.31	2.52	7.81	3.74	−0.65	0.520 −0.15
Impulse	7.03	2.44	6.42	1.96	−1.13	0.262 −0.28
Non-acceptance	12.86	6.45	11.28	3.99	−1.21	0.229 −0.29
Goals	7.55	3.82	6.97	2.68	−0.72	0.476 −0.17
Clarity	5.79	2.39	5.86	2.16	0.12	0.905 0.03
Strategies	5.62	2.48	5.36	2.21	−0.45	0.657 −0.11
Composite DERS^a^	47.17	16.16	43.69	12.36	−0.98	0.329 −0.24

*^a^DERS, Difficulties in Emotion Regulation Scale.*

#### Influence of Parental Emotional Regulation on School-Aged Children’s Emotional Regulation

A linear regression model was conducted to identify the putative predictor value of parental ER in school-aged children’s ER. The ERC composite was significantly correlated with parental DERS composite (*r* = 0.63, *p* < 0.001) and with all the subscales (Awareness *r* = 0.41, *p* < 0.05; Impulse *r* = 0.64, *p* < 0.001; Non-acceptance *r* = 0.39, *p* < 0.05; Goals *r* = 0.46, *p* < 0.0; Clarity *r* = 0.52, *p* < 0.01; and Strategies *r* = 0.42, *p* < 0.05). Regression results showed that parental ER, as assessed by the DERS composite score, predicted 38% of the variability of the children’s ERC composite score (*F* = 19.26, *p* < 0.001). That is, higher values in parental DERS composite score predicted higher scores in children’s ERC composite. When parents’ DERS subscales were included in the model, using a forward selection method, only the parent’s Impulse subscale was retained, explaining 54% of the variability of children’s ER measured by ERC composite score (*F* = 35.82, *p* < 0.001). The other subscales of parent’s DERS (awareness, non-acceptance, goals, clarity, and strategies) were excluded from the model (see Table 9). In both significant models, all the assumptions for regression analyses were satisfied including independence of errors, homoscedasticity, and normality of the distribution of residuals.

**TABLE 9:** Regression model: influences of parent’s emotional regulation on children and adolescent’s emotional regulation (Study 3).

	Composite DERS parents		
Variable	St. β	*t*	CI
Composite ERC children	0.619 *R*^2^ = 0.383	4.389***	0.160, 0.437
	Impulse DERS parents		
Composite ERC children^a^	0.732 *R*^2^ = 0.536	5.985***	1.656, 3.369
	Goals DERS parents		
Composite DERS adolescents°	0.353	2.069*	0.020, 3.092
	*R*^2^ = 0.125		

*ERC, emotion regulation checklist; DERS, Difficulties in Emotion Regulation Scale.*

*^a^Forward selection excluded awareness, non-acceptance, goals, clarity and strategies from the model.*

*°Forward selection excluded awareness, impulse, non-acceptance, clarity and strategies from the model.*

*p ≤ 0.05; ***p < 0.001.

#### Influence of Parental Emotional Regulation on Adolescents’ Emotional Regulation

A linear regression was conducted to identify the putative predictor variable of parental ER on the ER of adolescents. Adolescent DERS composite was significantly correlated with parental DERS composite (*r* = 0.36, *p* < 0.05) and with three parental DERS subscales (Goals *r* = 0.44, *p* < 0.05; Clarity *r* = 0.52, *p* < 0.01 and Strategies *r* = 0.38, *p* < 0.05). Parental ER assessed *via* DERS composite score did not conform a good predictor for the adolescent’s DERS composite score (*F* = 3.60, *p* = *0.067*). When parental DERS subscale scores were included in the model, the forward selection indicated that the parental Goal subscale showed a positive influence on the adolescent’s DERS composite score (*F* = 4.28, *p* = *0.047*). Specifically, the DERS’ Goal subscale explained 12.5% of the variability of adolescent’s ER assessed by the DERS composite score. The other subscales (awareness, impulse, non-acceptance clarity and strategies) were excluded from the model because they did not explain variability (see Table 9). Concerning the conditions of application, despite independence of errors, and homoscedasticity assumptions being satisfied, caution is advised when interpreting this result, since the subsequent analysis indicated that residuals did not follow normal distribution.

## Discussion

In the three present studies we investigated emotional regulation (ER) abilities in DLD populations compared to TD populations, by focusing on different developmental stages, different language abilities, and by using validated surveys. The first aim was to explore possible differences in ER in school-aged children and adolescent populations with and without DLD. The second and third aims were to investigate whether children’s and adolescents’ receptive and expressive vocabulary knowledge predicted ER 4 years later. Finally, we aimed to study possible differences between the ER of the parents of DLD populations compared to parents of TD populations, and whether parental ER predicts their children’s ER.

In study 1, results showed no significant differences between ER in school-aged children with DLD compared to TD children. The medium effect sizes in both ER and Lab subscales and the ERC composite score and the numerical differences (not statistically significant) between groups in the direction of our hypothesis (less ER in DLD group than TD group) suggest that possibly in larger samples we may find significantly less ER abilities in school-aged children with DLD compared to TD children. This trend is in line with findings by Fujiki et al. (2002, 2004). It is interesting, within this point, to go further and analyze individual items of the ERC survey to increase the understanding of those differences. Concerning the ER subscale, there were two items where the DLD group scored significantly lower than TD peers. First, the item that assesses empathetic ability (item 21) (i.e., the ability to capture emotions in others and to show the emotion to them). Lower empathetic ability can cause difficulties in getting close to others, which in turn makes it more difficult to establish social interactions. In this sense, our results seemed to converge with studies reporting social difficulties and lower quality of relationships with peers in children with DLD (Fujiki et al., 1999; Brinton and Fujiki, 2002; Andrés-Roqueta et al., 2016). Second, the item referring happy mood (item 1), showed that the DLD group scored lower in happiness than the TD group. This individual item’s results seem to coincide with results from other studies that show high levels of affective symptomatology, depression and anxiety in children with DLD (see Yew and O’Kearney, 2013 for review). In addition, when the Lab subscale was further analyzed, specifically, two items referring low tolerance to frustration and more temper tantrums (items 6 and 8), results showed higher scores in the DLD group compared to their typical peers. In this regard, it is possible that higher emotional activation along with the difficulty to understand their own emotional state and the emotional states of others could interfere in the ER strategies developed in children with DLD. However, these findings should be interpreted with caution due to the small sample size.

In addition, in study 1, the results also suggest that ER can be influenced by the expressive vocabulary knowledge that they had 4 years before. These results support the evidence that shows that a broader and richer vocabulary is a crucial factor in explaining differences in children’s regulation abilities. As suggested by Cole et al. (2010), and in line with recent studies that have shown that vocabulary scores were significantly positively associated with ER in early childhood (Vallotton and Ayoub, 2011; Ornaghi et al., 2019), our results indicate that expressive vocabulary is an important factor to take into account in other stages of childhood (around 7 years of age) and in the ER abilities developed later (around age 10). Thus, the first study of this project indicates that having a broad expressive vocabulary in school-aged children would help to clarify, understand, regulate, and express emotions property at later stages, specifically in children with DLD.

In study 2, results showed non-significant differences between ER in adolescents with and without DLD. Although numerically, not statistically, significant mean and median trends suggested lower levels of ER in adolescents with DLD, the effects sizes revealed that these differences were small, except for the Impulse subscale, where medium effect size was found. Taken together, results from study 1 and 2 showed that ER in population with DLD seems to improve in later developmental stages (i.e., adolescence) since the differences between DLD and TD groups appeared to be more prominent in school-aged children (study 1) than in adolescents (study 2). As far as we know, there are no previous studies analyzing ER in adolescents with DLD, hence our results should be interpreted with caution due to the small sample.

Regarding ER and vocabulary knowledge in adolescents in study 2, the regression models showed that none of the receptive or expressive vocabulary scores from wave 1 predicted any of the subscales or composite scores from the DERS survey 4 years later. Thus, taking into consideration results from study 1 and 2, we see that the relationship between vocabulary knowledge and ER differs at the two developmental stages analyzed. As we pointed out previously, expressive vocabulary knowledge at age 7 accounted for a significant amount of unique variance in ER around age 11, but neither receptive nor expressive vocabulary in children around 10-years-old predicted later ER abilities around age 15.

To sum up, the present results suggest that expressive vocabulary knowledge in school-aged children (study 1) is still important for their later ER abilities, in agreement with studies involving babies and toddlers (Vallotton and Ayoub, 2011; Ornaghi et al., 2019). In adolescence, however, this longitudinal relationship does not seem to hold.

The question remains whether other language abilities, apart from expressive vocabulary knowledge, in school-age children around 10-years-old, would better mediate with later ER abilities in adolescence. It seems that the capacity to use vocabulary verbally is not the core linguistic ability that regulates emotions in adolescence, perhaps because the emotions that are expressed at these ages have a greater degree of complexity and the measure of vocabulary, in isolation, is not informative enough in the prediction model. Thus, other language strategies should be analyzed apart from vocabulary to determine which ones help more in the regulation of emotions at this stage of development. Another question that the present results add to the discussion is the lack of predictivity of receptive knowledge on ER abilities in both samples (school-aged children and adolescents). Previous studies have shown that receptive vocabulary (with literacy as a mediator variable) in infancy is related to emotional competence (Westrupp et al., 2020). Future studies will need to assess receptive vocabulary as a possible prediction in later ER in children at the same developmental stage.

Finally, as far as we are concerned, this is the first study to examine differences between families with a child with DLD and families with TD children. Also, the examination of the impact of parents’ ER on the ER of their sons and daughters (with and without DLD), as we did in study 3, is new. Contrary to what we expected, results showed that there were no significant differences between any of the ER scores for the two groups of parents. Despite the emotional burden found in mothers of children with language disorders compared to mothers of TD children in past studies such as Lisa et al. (2019) and Ash et al. (2020), our data did not suggest more difficulties in ER abilities in parents of a child with DLD in comparison to a parent with a child with normal language abilities. More studies including parents with a child with DLD are needed to explore their ER and to be compared with our data.

Moreover, regression results in study 3 showed that the ER of the parents predicted the ER of their school-aged children. That is, parents with more ER difficulties reported more ER difficulties in their child. Specifically, parents’ Impulse subscale (i.e., feeling overwhelmed by emotions) seemed to be relevant during ER in middle childhood, specifically, it explained up to 54% of their children’s ER. Interestingly, this impact seemed to diminish significantly when the children grew up. That is to say, it seems that parental ER has a broader impact on their school-aged children than the impact parents’ ER has on adolescents, where it seems the relationship is more restricted. Our results converge with results derived from Tammilehto et al. (2021) who found that, while parental autonomy and intimacy with their middle-childhood sons and daughters had an impact on the children’s ER, this effect was not maintained long-term on adolescents. This pattern of no-long-term effect from parenting on adolescent ER is similar to the results in our study. Additionally, recent results pointed out a relevant protective factor of parental involvement in the education process of children with anxiety-depressive symptomatology and school problems (Valera-Pozo et al., 2020). Taken together, all these results seem to indicate that it would be important to broaden the focus of research into children and adolescents with DLD to include their parents, not only as informants, but as a part of a complex and comprehensive intervention.

Although it was not a core aim of the present study, we were interested to discover possible sex differences in ER in the different samples because different studies had previously shown that women presented higher ER during their lifespan than men (Nolen-Hoeksema, 2012 for review). Results from the three studies conducted on three different samples (children, adolescents, and parents), contrary to previous results, showed no sex differences in ER. It is worth considering that the children and adolescents’ samples were not equally compensated in number of girls and boys and the results may not reflect the actual differences. Thus, in future studies it will be necessary to represent both sexes equally in number to have more robust results. In study 3, that differed from the other two studies, focusing on parents’ ER, women were overrepresented. We asked families to choose which parent would answer the ER self-evaluation questionnaire, and most answers were from mothers (83%) versus 17% that were fathers. This unbalanced representation may be explained by the fact that mothers take more charge of caregiving matters at home. Taken together, the sex representation in future studies in children and parents will need to consider the aim of achieving the “Fix the Numbers” strategic approach proposed by the Gender Innovation project (Schiebinger et al., 2011) to ensure sex equality by increasing underrepresented girls’ participation and also to ensure mothers and fathers representation when caregiving is one of the variables considered.

### Limitations, Future Research Directions, and Educational Implications

This paper is not without its limitations. First, due to the well-known complexity involved in recruiting clinical population, the samples in the first and second study are smaller than desirable, and results can be interpreted as informative and not as a robust conclusion due to the preliminary nature of the data. Thus, for the two first studies caution is needed in generalizing these results to all the DLD population. Further studies with more participants need to contrast our results. Also, in study 1, data collection relied on parental report measures, with the associated risk of bias and subjectivity. In the future, it would be desirable to combine parent reports or self-reported measures with more ecological instruments, such as observation grids designed to collect information about children’ ER. Furthermore, previous research has demonstrated that the use of ER strategies can vary by sociocultural context (Rychlowska et al., 2015). The cultural values related to emotions provide manners to use emotions in order to facilitate norms for ER and interpersonal relationships. For example, research on cultural differences in ER has demonstrated that individuals in Eastern cultures tend to use suppression (i.e., an ER strategy characterized by controlling or neutralizing emotional behavior) more frequently than those in Western cultures (Matsumoto et al., 2008). Although, in the present study we did not examine the interference of sociocultural variables in ER it is important to highlight that these aspects could be fundamental for the replication of the study in other cultures. Despite these limitations, a powerful factor of the present study is the longitudinal design that enabled us to identify predictive relationships between vocabulary and ER, adding to the field more understanding of the contribution of intra-individual language abilities to explaining variance in children’s ER. Thus, the present project highlights the key role of expressive vocabulary in explaining ER in childhood. It also highlights that this prediction does not translate to adolescence. Intervention implications in this regard underscore the stimulation and strengthening of school-aged children’s ability to label words, actions and feeling as a strategy to prevent difficulties in later ER. It remains unclear yet which language ability would be most important to reinforce in later primary school ages to continue preventing ER difficulties in adolescence. The present results also shed light on the ER abilities of the fathers and mothers of children with and without DLD and the relationship of those abilities with their children’s ER, which should encourage educators and speech language pathologists to include parents’ assessments in the holistic evaluation of, and intervention in, children with language and ER difficulties.

In summary, the present preliminary data are consistent with the claim that language, and specifically expressive vocabulary, holds a fundamental role in ER, at least during primary school years. These data also add new evidence about the impact of parents’ ER on their children’s ER. According to our findings, the ER of parents of school-aged children (but not of adolescents) predicts their children’ ER.

## Data Availability Statement

The raw data supporting the conclusions of this article will be made available by the authors, without undue reservation, to any qualified researcher.

## Ethics Statement

The studies involving human participants were reviewed and approved by the Universitat Oberta de Catalunya (UOC) Ethics Committee. Written informed consent to participate in this study was provided by the participants’ legal guardian/next of kin.

## Author Contributions

MA and NA contributed to conception and design of the study, organized the database, performed the statistical analysis, wrote the first draft of the manuscript, and wrote sections of the manuscript. NE-G contributed to the statistical analysis and participated in the written sections of the manuscripts. LF contributed to the data collection and curation. MS-T and LA supervised and administrated the project and provided the funding acquisition. All authors contributed to manuscript revision, read, and approved the submitted version.

## Conflict of Interest

The authors declare that the research was conducted in the absence of any commercial or financial relationships that could be construed as a potential conflict of interest.

## Publisher’s Note

All claims expressed in this article are solely those of the authors and do not necessarily represent those of their affiliated organizations, or those of the publisher, the editors and the reviewers. Any product that may be evaluated in this article, or claim that may be made by its manufacturer, is not guaranteed or endorsed by the publisher.

## References

[B1] Adani F. Forgiarini M. Guasti M. T. Van Der Lely H. K. J. (2014). Number dissimilarities facilitate the comprehension of relative clauses in children with (Grammatical) specific language impairment. *J. Child Lang.* 41 811–841. 10.1017/S0305000913000184 23806292 PMC4068306

[B2] Aguilar-Mediavilla E. Buil-Legaz L. López-Penadés R. Sanchez-Azanza V. A. Adrover-Roig D. (2019). Academic outcomes in bilingual children with developmental language disorder: a longitudinal study. *Front. Psychol.* 10:531. 10.3389/fpsyg.2019.00531 PMC6421289 30915007

[B3] Ahufinger N. Guerra E. Ferinu L. Andreu L. Sanz-Torrent M. (2021). Cross-situational statistical learning in children with developmental language disorder. *Lang. Cogn. Neurosci.* 36 1180–1200. 10.1080/23273798.2021.1922723

[B4] Alt M. Plante E. (2006). Factors that influence lexical and semantic fast mapping of young children with specific language impairment. *J. Speech Lang. Hear Res.* 49 941–954. 10.1044/1092-4388(2006/068) 17077207

[B5] American National Standard Institute (1997). *American National Standard: Methods for Calculation of the Speech Intelligibility Index.* New York, NY: Acoustical Society of America.

[B6] Andrés-Roqueta C. Adrian J. E. Clemente R. A. Villanueva L. (2016). Social cognition makes an independent contribution to peer relations in children with specific language impairment. *Res. Dev. Disabil.* 4 277–290. 10.1016/j.ridd.2015.12.015 26745788

[B7] Ash A. C. Christopulos T. T. Redmond S. M. (2020). “Tell me about your child”: a grounded theory study of mothers’ understanding of language disorder. *Am. J. Speech Lang. Pathol.* 29 819–840. 10.1044/2020_AJSLP-19-00064 32348158 PMC7842869

[B8] Baker S. (2018). “The effects of parenting on emotion and self-regulation,” in *Handbook of Parenting and Child Development*, eds Sanders A. Morawska M. R. (Berlin: Springer), 217–240. 10.1007/978-3-319-94598-9_10

[B9] Barrett L. F. Lindquist K. A. Gendron M. (2007). Language as context for the perception of emotion. *Trends Cogn. Sci.* 11 327–332. 17625952 10.1016/j.tics.2007.06.003 PMC2225544

[B10] Beck L. Kumschick I. R. Eid M. Klann-Delius G. (2012). Relationship between language competence and emotional competence in middle childhood. *Emotion* 12, 503–514. 10.1037/A0026320 22148995

[B11] Beitchman J. H. Wilson B. Brownlie E. B. Walters H. Lancee W. (1996). Long-term consistency in speech/language profiles: i. developmental and academic outcomes. *J. Am. Acad. Child Adolesc. Psychiatry* 35 804–814. 10.1097/00004583-199606000-00021 8682762

[B12] Beitchman J. H. Wilson B. Johnson C. J. Atkinson L. Young A. Adlaf E. (2001). Fourteen-year follow-up of speech/language-impaired and control children: psychiatric outcome. *J. Am. Acad. Child Adolesc. Psychiatry* 40 75–82. 10.1097/00004583-200101000-00019 11195567

[B13] Bishop D. V. M. Snowling M. J. Thompson P. A. Greenhalgh T. Adams C. Archibald L. (2016). CATALISE: a multinational and multidisciplinary Delphi consensus study. Identifying language impairments in children. *PLoS One* 11:e0158753. 10.1371/journal.pone.0158753 27392128 PMC4938414

[B14] Bishop D. V. M. Snowling M. J. Thompson P. A. Greenhalgh T. Adams C. Archibald L. (2017). Phase 2 of CATALISE: a multinational and multidisciplinary Delphi consensus study of problems with language development: terminology. *J. Child Psychol. Psychiatry Allied Disciplines* 58 1068–1080. 10.1111/jcpp.12721 28369935 PMC5638113

[B15] Bosacki S. L. Moore C. (2004). Preschoolers’ understanding of simple and complex emotions: links with gender and language. *Sex Roles* 50 659–675. 10.1023/b:sers.0000027568.26966.27

[B16] Botting N. Durkin K. Toseeb U. Pickles A. Conti-Ramsden G. (2016). Emotional health, support, and self-efficacy in young adults with a history of language impairment. *Br. J. Dev. Psychol.* 34 538–554. 10.1111/bjdp.12148 27226087 PMC5082521

[B17] Brinton B. Fujiki M. (2002). “Social development in children with specific language impairment and profound hearing loss,” in *Handbook of Childhood Social Development*, eds Smith P. Hart C. (Hoboken, NJ: Blackwell Publishing Inc), 588–603.

[B18] Catts H. Bridges M. Little T. Tomblin J. (2008). Reading achievement growth in children with language impairments. *J. Speech Lang Hear. Res.* 51 1569–1579. 18695010 10.1044/1092-4388(2008/07-0259) PMC3763805

[B19] Cole P. M. Cohen L. H. (2009). Preschoolers’ emotion regulation strategy understanding: relations with emotion socialization and child self-regulation. *Wiley Online Library* 18 324–352.

[B20] Cole P. M. Armstrong L. M. Pemberton C. K. (2010). “The role of language in the development of emotion regulation,” in *Child Development at the Intersection of Emotion and Cognition*, eds Calkins D. Bell M. A. (Washington, DC: American Psychological Association).

[B21] Conti-Ramsden G. Botting N. (2008). Emotional health in adolescents with and without a history of specific language impairment (SLI). *J. Child Psychol. Psychiatry Allied Disciplines* 49 516–525. 10.1111/j.1469-7610.2007.01858.x 18221347

[B22] Conti-Ramsden G. Mok P. L. H. Pickles A. Durkin K. (2013). Adolescents with a history of specific language impairment (SLI): strengths and difficulties in social, emotional and behavioral functioning. *Res. Dev. Disabil.* 34 4161–4169. 10.1016/j.ridd.2013.08.043 24077068 PMC3830176

[B23] Conti-Ramsden G. Mok P. Durkin K. Pickles A. Toseeb U. Botting N. (2019). Do emotional difficulties and peer problems occur together from childhood to adolescence? the case of children with a history of developmental language disorder (DLD). *Eur. Child Adolesc. Psychiatry* 28 993–1004. 10.1007/s00787-018-1261-6 30519863 PMC6647450

[B24] Cutting A. L. Dunn J. (1999). Theory of Mind, emotion understanding, language, and family background: individual differences and interrelations. *Child Dev.* 70 853–865. 10.1111/1467-8624.00061 10446724

[B25] Denham S. A. (1998). *Emotional Development in Young Children.* New York NY: Guilford Press.

[B26] Downs A. Strand P. Cerna S. (2007). Emotion understaanding in English- and Spanish-speaking preschoolers enrolled in head start. *Soc. Dev.* 16 410–439. 10.1111/j.1467-9507.2007.00391.x

[B27] Dunn L. M. Dunn L. M. Arribas D. (2006). *Test de Vocabulario en Imágenes (PPVT-III PEABODY).* Madrid: TEA Ediciones.

[B28] Durkin K. Conti-Ramsden G. (2007). Language, social behavior, and the quality of friendships in adolescents with and without a history of specific language impairment. *Child Dev.* 78 1441–1457. 10.1111/j.1467-8624.2007.01076.x 17883441

[B29] Durkin K. Conti-Ramsden G. (2010). Young people with specific language impairment: a review of social and emotional functioning in adolescence. *Child Lang. Teach. Ther.* 26 105–121. 10.1177/0265659010368750

[B30] Eisenberg N. Champion C. Ma Y. (2004). Emotion-related regulation: an emerging construct. *Merrill Palmer Q.* 50 236–259. 10.1353/mpq.2004.0016 34409987

[B31] Eisenberg N. Sadovsky A. Spinrad T. L. (2005). Associations of emotion-related regulation with language skills, emotion knowledge, and academic outcomes. *New Dir. Child Adolesc. Dev.* 109 109–118. 10.1002/cd.143 16342899 PMC1361289

[B32] Eyer J. A. Leonard L. B. (1995). Functional categories and specific language impairment: a case study. *Lang. Acquis.* 4 177–203. 10.1207/s15327817la0403_1

[B33] Forrest C. L. Gibson J. L. St Clair M. C. (2021). Emotional problems in adolescents. *Int. J. Environ. Res. Public Health* 18:1221. 10.3390/ijerph18031221 PMC7908163 33572993

[B34] Forrest C. L. Gibson J. L. Halligan S. L. St Clair M. C. (2020). A cross-lagged analysis of emotion regulation, peer problems, and emotional problems in children with and without early language difficulties: evidence from the millennium cohort study. *J. Speech Lang. Hear Res.* 63 1227–1239. 10.1044/2020_JSLHR-19-00188 32315250

[B35] Fujiki M. Brinton B. Clarke D. (2002). Emotion regulation in children with specific language impairment. *Lang. Speech Hear. Serv. Schl.* 33 102–111. 10.1044/0161-1461(2002/008) 27764463

[B36] Fujiki M. Brinton B. Hart C. H. Fitzgerald A. H. (1999). Peer acceptance and friendship in children with specific language impairment. *Top. Lang. Disord.* 19 34–48. 10.1044/1092-4388(2004/012)

[B37] Fujiki M. Spackman M. P. Brinton B. Hall A. (2004). The relationship of language and emotion regulation skills to reticence in children with specific language impairment. *J. Speech Lang. Hear Res.* 47 637–646. 10.1044/1092-4388(2004/049) 15212574

[B38] Girme Y. U. Jones R. E. Fleck C. Simpson J. A. Overall N. C. (2020). Emotion Infants’. attachment insecurity predicts attachment-relevant emotion regulation strategies in adulthood. *Emotion* 21 260–272. 10.1037/emo0000721 31916790 PMC7343591

[B39] Gómez-Simón I. Penelo E. de la Osa N. (2014). Estructura factorial e invariancia de la Escala de Dificultades en la Regulación Emocional (DERS) en adolescentes españoles. *Psicothema* 26 401–408. 25069562 10.7334/psicothema2013.324

[B40] Goubet K. E. Chrysikou E. G. (2019). Emotion regulation flexibility: gender differences in context sensitivity and repertoire. *Front. Psychol.* 10:935. 10.3389/fpsyg.2019.00935 31143142 PMC6521736

[B41] Gratz K. L. Roemer L. (2004). Multidimensional assessment of emotion regulation and dysregulation: development, factor structure, and initial validation of the difficulties in emotion regulation scale. *J. Psychopathol. Behav. Assess.* 26 41–54. 10.1023/b:joba.0000007455.08539.94

[B42] Gray S. (2004). Word learning by preschoolers with specific language impairment: predictors and poor learners. *J. Speech Lang. Hear Res.* 47 1117–1132. 10.1044/1092-4388(2004/083) 15603466

[B43] Gray S. Plante E. Vance R. Henrichsen M. (1999). The diagnostic accuracy of four vocabulary tests administered to preschool-age children. *Lang. Speech Hear. Serv. Schl.* 30 196–206. 10.1044/0161-1461.3002.196 27764281

[B44] Gross J. J. (2015a). “Conceptual and empirical foundations,” in *Handbook of Emotion Regulation*, ed. Gross J. J. (New York. NY: The Guilford Press).

[B45] Gross J. J. (2015b). Emotion regulation: current status and future prospects. *Psychol. Inquiry* 26 1–26. 10.1254/fpj.151.21 29321392

[B46] Hervás G. Jódar R. (2008). The Spanish version of the difficulties in emotion regulation scale. *Clin. Salud* 19 139–156.

[B47] Hilt L. M. Armstrong J. M. Essex M. J. (2012). Early family context and development of adolescent ruminative style: moderation by temperament. *Cogn. Emot.* 26 916–926. 10.1080/02699931.2011.621932 22077906 PMC3292851

[B48] Jackson E. Leitão S. Claessen M. Boyes M. (2019). The evaluation of word-learning abilities in people with developmental language disorder: a scoping review. *Int. J. Lang. Commun. Disord.* 54 742–755. 10.1111/1460-6984.12490 31276299

[B49] Kapa L. L. Plante E. (2015). Executive function in SLI: recent advances and future directions. *Curr. Dev. Disord. Rep.* 2 245–252. 10.1007/s40474-015-0050-x 26543795 PMC4629777

[B50] Kaufman A. S. Kaufman N. L. (1990). *Test Breve de Inteligencia Kaufman (K-BIT).* London: Pearson.

[B51] Kaufman A. S. Kaufman N. L. (2004). *K-Bit, Test breve de inteligen- cia Kaufman.* London: Pearson.

[B52] Köckeritz M. Klinkhammer J. Salisch M. (2010). Die Entwicklung des Emotionswissens und der behavioralen selbstregulation bei vorschulkindern mit und ohne Migrationshintergrund. *Praxis der Kinderpsychologie und Kinderpsychiatrie* 59 529–544. 10.13109/prkk.2010.59.7.529 20957775

[B53] Kopp C. B. (1989). Regulation of distress and negative emotions: a developmental view. *Dev. Psychol.* 25, 343–354. 10.1037/0012-1649.25.3.343

[B54] Kopp C. B. (1992). “Emotional distress and control in young children,” in *Emotion and its Regulation in Early Development: New Directions in Child Development*, eds Eisenberg N. Fabes R. A. (San Francisco, CA: Jossey-Bass), 41–56. 10.1002/cd.23219925505 1608514

[B55] Lee J. C. Tomblin J. B. (2012). Reinforcement learning in young adults with developmental language impairment. *Brain Lang.* 123 154–163. 22921956 10.1016/j.bandl.2012.07.009 PMC3502713

[B56] Leonard L. B. (1995). Functional categories in the grammars of children with specific language impairment. *J. Speech Hear. Res.* 38 1270–1283. 10.1044/jshr.3806.1270 8747820

[B57] Leonard L. B. (2014). *Children with Specific Language Impairment*, 2nd Edn. Cambridge, MA: MIT Press.

[B58] Lisa R. Pola R. Franz P. Jessica M. (2019). Developmental language disorder: maternal stress level and behavioural difficulties of children with expressive and mixed receptive-expressive DLD. *J. Commun. Disord.* 80 1–10. 10.1016/j.jcomdis.2019.03.006 30999162

[B59] Matsumoto D. Yoo S. H. Nakagawa S. Alexandre J. Altarriba J. Anguas-Wong A. M. (2008). Culture. emotion regulation, and adjustment. *J. Pers. Soc. Psychol.* 94 925–937. 18505309 10.1037/0022-3514.94.6.925

[B60] Morris A. S. Silk J. S. Steinberg L. Myers S. S. Robinson L. R. (2007). The role of the family context in the development of emotion regulation. *Soc. Dev.* 16 361–388. 10.1111/j.1467-9507.2007.00389.x 19756175 PMC2743505

[B61] Moscati V. Rizzi L. Vottari I. Chilosi A. M. Salvsdorini R. Gusati A. T. (2020). Morphosyntactic weaknesses in developmental language disorder: the role of structure and agreement configurations. *J. Child Lang.* 47 909–944. 10.1017/S0305000919000709 31957622

[B62] Nolen-Hoeksema S. (2012). Emotion regulation and psychopathology: the role of gender. *Annu. Rev. Clin. Psychol.* 8 161–187. 22035243 10.1146/annurev-clinpsy-032511-143109

[B63] Norbury C. F. Gooch D. Wray C. Baird G. Charman T. Simonoff E. (2016). The impact of nonverbal ability on prevalence and clinical presentation of language disorder: evidence from a population study. *J. Child Psychol. Psychiatry Allied Disciplines* 57 1247–1257. 10.1111/jcpp.12573 27184709 PMC5082564

[B64] Ornaghi V. Pepe A. Agliati A. Grazzani I. (2019). The contribution of emotion knowledge, language ability, and maternal emotion socialization style to explaining toddlers’ emotion regulation. *Soc. Dev.* 28 581–598. 10.1111/sode.12351

[B65] Pons F. Lawson J. Harris P. L. de Rosnay M. (2003). Individual differences in children’s emotion understanding: effects of age and language. *Scand. J. Psychol.* 44 347–353. 10.1111/1467-9450.00354 12887556

[B66] Redmond S. Rice M. (2002). Stability of behavioral ratings of children with SLI. *J. Speech Lang. Hear. Res.* 45 190–201. 14748648 10.1044/1092-4388(2002/014)

[B67] Rice M. L. Buhr J. C. Nemeth M. (1990). Fast mapping word-learning abilities of language-delayed preschoolers. *J. Speech Hear. Disord.* 55 33–42. 2299838 10.1044/jshd.5501.33

[B68] Rice M. L. Oetting J. B. Marquis J. Bode J. Pae S. (1994). Frequency of input effects on word comprehension of children with specific language impairment. *J. Speech Hear. Res.* 37 106–122. 10.1044/jshr.3701.106 8170118

[B69] Rychlowska M. Miyamoto Y. Matsumoto D. Hess U. Gilboa-Schechtman E. Kamble S. (2015). Heterogeneity of long-history migration explains cultural differences in reports of emotional expressivity and the functions of smiles. *Proc. Natl. Acad. Sci. USA* 112 E2429–E2436. 10.1073/pnas.1413661112 25902500 PMC4434713

[B70] Saarni C. (1999). *The Development of Emocional Competence.* New York, NY: Guilford Press.

[B71] Sarmento-Henrique R. Lucas-Molina B. Quintanilla-Cobián L. Giménez-Dasí M. (2017). La evaluación multi-informante de la regulación emocional en edad preescolar: un estudio longitudinal. *Psicol. Educ.* 23 1–7. 10.1016/j.pse.2017.01.001

[B72] Schiebinger L. Klinge I. Sánchez de Madariaga I. Paik H. Y. Schraudner M. (2011). *Gendered Innovations in Science, Health & Medicine, Engineering, and Environment.* Available Online at: http://genderedinnovations.stanford.edu/how_to_cite.html (accessed January 21, 2015).

[B73] Semel E. Wiig E. H. Secord W. A. (2006). *Spanish Clinical Evaluation of Language Fundamentals-4 (CELF-4).* London: Pearson.

[B74] Shields A. Cicchetti D. (1997). Emotion regulation among school-age children: the development and validation of a new criterion Q-sort scale. *Dev. Psychol.* 33 906–916. 10.1037//0012-1649.33.6.906 9383613

[B75] St Clair M. C. Forrest C. L. Yew S. G. K. Gibson J. L. (2019). Early risk factors and emotional difficulties in children at risk of developmental language disorder: a population cohort study. *J. Speech Lang. Hear. Res.* 62 2750–2771. 31306586 10.1044/2018_JSLHR-L-18-0061

[B76] St Clair M. C. Pickles A. Durkin K. Conti-Ramsden G. (2011). A longitudinal study of behavioral, emotional and social difficulties in individuals with a history of specific language impairment (SLI). *J. Commun. Disord.* 44 186–199. 10.1016/j.jcomdis.2010.09.004 20970811

[B77] Tager-Flusberg H. Cooper J. (1999). Present and future possibilities for defining a phenotype for specific language impairment. *J. Speech Lang. Hear. Res.* 42 1275–1278. 10515521 10.1044/jslhr.4205.1275

[B78] Tammilehto J. Punamäki R. L. Flykt M. Vänskä M. Heikkilä L. M. Lipsanen J. (2021). Developmental stage-specific effects of parenting on adolescents’ emotion regulation: a longitudinal study from infancy to late adolescence. *Front. Psychol.* 12:582770. 10.3389/fpsyg.2021.582770 34149494 PMC8211896

[B79] Thompson R. A. (1991). Emotional regulation and emotional development. *Educ. Psychol. Rev.* 3 269–307.

[B80] Tomblin J. B. Freese P. R. Records N. L. (1992). Diagnosing specific language impairment in adults for the purpose of pedigree analysis. *J. Speech Hear. Res.* 35 832–843. 10.1044/jshr.3504.832 1405540

[B81] Valera-Pozo M. Adrover-Roig D. Pérez-Castelló J. A. Sanchez-Azanza V. A. Aguilar-Mediavilla E. (2020). Behavioral, emotional and school adjustment in adolescents with and without Developmental Language Disorder (DLD) is related to family involvement. *Int. J. Environ. Res. Public Health* 17:1949. 10.3390/ijerph17061949 32188170 PMC7142754

[B82] Vallotton C. Ayoub C. (2011). Use your words: the role of language in the development of toddlers’ self-regulation. *Early Child. Res. Q.* 26 169–181. 10.1016/j.ecresq.2010.09.002 21969766 PMC3184006

[B83] van den Bedem N. P. Dockrell J. E. van Alphen P. M. de Rooij M. Samson A. C. Harjunen E. L. (2018). Depressive symptoms and emotion regulation strategies in children with and without developmental language disorder: a longitudinal study. *Int. J. Lang. Commun. Disord.* 53 1110–1123. 10.1111/1460-6984.12423 30141224 PMC6282613

[B84] Van Der Lely H. K. J. (1998). SLI in children: movement, economy, and deficits in the computational-syntactic system. *Lang. Acquis.* 7 161–192. 10.1207/s15327817la0702-4_4

[B85] Van Der Lely H. K. J. Rosen S. Adlard A. (2004). Grammatical language impairment and the specificity of cognitive domains: relations between auditory and language abilities. *Cognition* 94 167–183. 10.1016/j.cognition.2004.01.003 15582625

[B86] Van Lissa C. J. Keizer R. Van Lier P. A. C. Meeus W. H. J. Branje S. (2019). The role of fathers’ versus mothers’ parenting in emotion-regulation development from mid-late adolescence: disentangling between-family differences from within-family effects. *Dev. Psychol.* 55 377–389. 10.1037/dev0000612 30570297

[B87] Von Suchodoletz W. Macharey G. (2006). Stigmatisierung sprachgestörter kinder aus sicht der eltern. *Prax. Kinderpsychol. Kinderpsychiatr.* 55, 711–723. 17212189

[B88] Westrupp E. M. Reilly S. McKean C. Law J. Mensah F. Nicholson J. M. (2020). Vocabulary development and trajectories of behavioral and emotional difficulties *via* academic ability and peer problems. *Child Dev.* 91 e365–e382. 10.1111/cdev.13219 30697706

[B89] WMA (2013). World medical association declaration of helsinki ethical principles for medical research involving human subjects. *JAMA* 310 2191–2194. 10.1001/jama.2013.281053 24141714

[B90] Yew S. G. K. O’Kearney R. (2013). Emotional and behavioural outcomes later in childhood and adolescence for children with specific language impairments: meta-analyses of controlled prospective studies. *J. Child Psychol. Psychiatry Allied Disciplines* 54 516–524. 10.1111/jcpp.12009 23082773

