# Supplementary material for: Vocabulary Abilities and Parents’ Emotional Regulation Predict Emotional Regulation in School-Age Children but Not Adolescents With and Without Developmental Language Disorder
Source: Front Psychol. 2021 Dec 9;12:748283. doi: 10.3389/fpsyg.2021.748283 (PMC8695603; doi:10.3389/fpsyg.2021.748283)
Supplement: Supplementary file 1 [file Data_Sheet_1.docx]

| **Supplementary Table S1**. *Sex differences concerning emotional regulation comparing within DLD group -8 boys and 5 girls- and within TD group -14 boys and 6 girls- (Study 1)* | | | | | | | | | | | | | |
| --- | --- | --- | --- | --- | --- | --- | --- | --- | --- | --- | --- | --- | --- |
|  |  | **Boys** | | |  | **Girls** | | |  | **Comparison** | |  |  |
|  | **Variable** | **Mean** | **SD** | **Median MAD** |  | **Mean** | **SD** | **Mean MAD** |  | **Zª** | ***Effect size*** | |  |
| DLD | ER subscale | 25.13 | 4.55 | 26.00 4.00 |  | 24.60 | 3.78 | 23.00 2.00 |  | -.07 | -.03 | |  |
|  | Lab subscale | 29.63 | 6.44 | 29.00 4.5 |  | 29.20 | 6.22 | 27.00 1.00 |  | -.44 | .15 | |  |
|  | Composite ERC | 44.50 | 10.17 | 43.00 8.50 |  | 44.60 | 9.02 | 44.00 7.00 |  | -.15 | -.05 | |  |
| TD | ER subscale | 28.00 | 1.71 | 28.00 1.00 |  | 26.99 | 4.09 | 26.00 1.00 |  | -1.14 | .32 | |  |
|  | Lab subscale | 26.64 | 4.27 | 25.50 2.50 |  | 26.17 | 5.98 | 25.50 3.50 |  | -.25 | .07 | |  |
|  | Composite ERC | 38.64 | 5.33 | 38.00 4.00 |  | 40.17 | 8.47 | 38.00 6.00 |  | -.25 | -.07 | |  |
|  | ER subscale= Emotion Regulation Subscale from ERC, Lab Subscale= Lability/Negativity Subscale from ERC, ERC= Emotion Regulation Checklist.  *ªNon-parametric Mann-Whitney U test was performed; For the Mann-Whitney test, effect size is given by the rank biserial correlation.* | | | | | | | | | | | |  |

| **Supplementary Table S2**. *Sex differences concerning emotional regulation comparing within DLD adolescent group -9 boys and 7 girls- and within TD adolescent group -12 boys and 4 girls- (Study 2)* | | | | | | | | | | | |  |
| --- | --- | --- | --- | --- | --- | --- | --- | --- | --- | --- | --- | --- |
|  |  | **Boys** | | |  | **Girls** | | |  | **Comparison** | |  |
|  | **Variable** | **Mean** | **SD** | **Median MAD** |  | **Mean** | **SD** | **Median MAD** |  | **Zª** | ***Effect size*** |  |
| DLD | Awareness | 13.22 | 3.27 | 14.00 2.00 |  | 9.57 | 3.74 | 9.00 4.00 |  | -1.92 | .57 |  |
|  | Impulse | 8.88 | 4.31 | 8.00 2.00 |  | 9.29 | 5.56 | 8.00 3.00 |  | -0.22 | .06 |  |
|  | Non-Acceptance | 10.89 | 4.83 | 8.00 0.00 |  | 10.57 | 3.91 | 8.00 1.00 |  | -0.39 | .11 |  |
|  | Goals | 10.89 | 4.86 | 9.00 1.00 |  | 8.86 | 4.85 | 7.00 2.00 |  | -1.02 | .30 |  |
|  | Clarity | 7.56 | 2.40 | 7.00 1.00 |  | 8.86 | 4.38 | 6.00 1.00 |  | -0.05 | -.02 |  |
|  | Strategies | 5.33 | 1.80 | 4.00 0.00 |  | 5.71 | 1.79 | 5.00 1.00 |  | -0.66 | -.19 |  |
|  | Composite DERS | 56.78 | 14.88 | 52.00 7.00 |  | 52.85 | 16.68 | 50.00 17.00 |  | -0.79 | .24 |  |
| TD | Awareness | 9.58 | 2.81 | 9.50 1.50 |  | 11.25 | 2.36 | 12.00 1.00 |  | -1.10 | -0.38 |  |
|  | Impulse | 6.92 | 2.88 | 5.50 0.50 |  | 6.25 | 1.50 | 6.00 1.00 |  | -0.07 | 0.02 |  |
|  | Non- Acceptance | 9.58 | 2.64 | 9.00 2.00 |  | 11.75 | 2.87 | 12.50 1.50 |  | -1.35 | -0.46 |  |
|  | Goals | 7.50 | 2.54 | 7.00 1.00 |  | 9.00 | 3.74 | 8.50 2.00 |  | -0.86 | -0.29 |  |
|  | Clarity | 6.67 | 2.06 | 6.50 1.50 |  | 8.00 | 2.71 | 7.00 0.50 |  | -0.86 | -0.29 |  |
|  | Strategies | 5.42 | 2.02 | 5.00 1.00 |  | 6.00 | 1.15 | 6.00 1.00 |  | -1.27 | -0.42 |  |
|  | Composite DERS | 45.67 | 8.92 | 44.50 6.50 |  | 52.25 | 3.10 | 53.00 1.50 |  | -1.71 | -.58 |  |
|  | DERS= Difficulties in Emotion Regulation Scale  *ªNon-parametric Mann-Whitney U test was performed; For the Mann-Whitney test, effect size is given by the rank biserial correlation.* | | | | | | | | | | | |
